# Disruption of Vitamin D and Calcium Signaling in Keratinocytes Predisposes to Skin Cancer

**DOI:** 10.3389/fphys.2016.00296

**Published:** 2016-07-12

**Authors:** Daniel D. Bikle, Yan Jiang, Thai Nguyen, Yuko Oda, Chia-ling Tu

**Affiliations:** Departments of Medicine and Dermatology, VA Medical Center and University of California, San FranciscoSan Francisco, CA, USA

**Keywords:** vitamin D receptor, calcium sensing receptor, squamous cell carcinoma, long non coding RNA, UVB, hedgehog, β–catenin

## Abstract

1,25 dihydroxyvitamin D (1,25(OH)_2_D), the active metabolite of vitamin D, and calcium regulate epidermal differentiation. 1,25(OH)_2_D exerts its effects through the vitamin D receptor (VDR), a transcription factor in the nuclear hormone receptor family, whereas calcium acts through the calcium sensing receptor (Casr), a membrane bound member of the G protein coupled receptor family. We have developed mouse models in which the *Vdr* and *Casr* have been deleted in the epidermis (^epid^*Vdr*^−∕−^ and ^epid^*Casr*^−∕−^). Both genotypes show abnormalities in calcium induced epidermal differentiation *in vivo* and *in vitro*, associated with altered hedgehog (HH) and β–catenin signaling that when abnormally expressed lead to basal cell carcinomas (BCC) and trichofolliculomas, respectively. The *Vdr*^−∕−^ mice are susceptible to tumor formation following UVB or chemical carcinogen exposure. More recently we found that the keratinocytes from these mice over express long non-coding RNA (lncRNA) oncogenes such as H19 and under express lncRNA tumor suppressors such as lincRNA-21. Spontaneous tumors have not been observed in either the ^epid^*Vdr*^−∕−^ or ^epid^*Casr*^−∕−^. But in mice with epidermal specific deletion of both *Vdr* and *Casr* (^epid^*Vdr*^−∕−^/^epid^*Casr*^−∕−^ [DKO]) tumor formation occurs spontaneously when the DKO mice are placed on a low calcium diet. These results demonstrate important interactions between vitamin D and calcium signaling through their respective receptors that lead to cancer when these signals are disrupted. The roles of the β–catenin, hedgehog, and lncRNA pathways in predisposing the epidermis to tumor formation when vitamin D and calcium signaling are disrupted will be discussed.

## Introduction

Skin cancer is the most common form of cancer with an incidence estimated to be over 5 million skin cancers per year in the United States and rising (American Cancer Society, data available at http://www.cancer.org.cancer, April, 2016 update). Most of these (80%) are basal cell carcinomas (BCC). Squamous cell carcinomas (SCC) make up another 16% and melanomas another 4%. Sunlight, in particular the UVB part of the spectrum (up to 5% of the UV light reaching earth depending on the zenith angle of the sun associated with time of day and season), is the major etiologic agent for these cancers. UVB (280–320 nm) is absorbed by DNA in the epidermis resulting in mutations identified as cyclobutane pyrimidine dimers (CPDs) and pyrimidine (6–4) pyrimidone photoproducts (6–4 PP) that lead to C to T or CC to TT mutations if not repaired. These are commonly referred to as the UVB “signature” lesion (Freeman et al., 1989; Hussein, 2005). On the other hand UVA (320–400 nm), comprising 95% of the UV light reaching earth, is capable of penetrating into the dermis and does its DNA damage primarily by oxidative processes (e.g., 8 hydroxy 2′ deoxyguanosine production), but CPDs may be produced at higher levels (Besaratinia et al., 2005). On the other hand UVB is required for vitamin D_3_ production converting 7-dehydrocholesterol levels in the skin to pre vitamin D_3_, which then isomerizes to vitamin D_3_. White males with class III pigmentation require 18 mJ/cm^2^ UVB exposure for vitamin D_3_ production, (Matsuoka et al., 1989), a dose not likely to lead to tumors in most individuals. However, the efficiency of vitamin D production by solar radiation is less than that of monochromatic UVB in part due to the UVA portion of solar radiation that influences the ratio of pre D_3_ to lumisterol_3_ produced and that may contribute to tumor formation over and above that of UVB (MacLaughlin et al., 1982; Agar et al., 2004). Vitamin D_3_ production is not the only metabolic step of which the skin is capable. Both the further conversion to 25OHD_3_ (via Cyp27A1 and Cyp2R1) and then to its active metabolite 1,25(OH)_2_D_3_ (via Cyp27b1) (Bikle et al., 1986) take place. This latter step is promoted by UVR (Muthusamy and Piva, 2009), perhaps by stimulation of the expression of cytokines such as TNF (Bikle et al., 1991). Moreover the vitamin D receptor (VDR) is expressed in both melanocytes (Colston et al., 1982) and keratinocytes (Pillai et al., 1988) and have been shown to respond to 1,25(OH)_2_D_3_ by a reduction in proliferation and promotion of differentiation (Colston et al., 1981; Bikle, 2012b). A key rationale for our studies in the protective role of calcium and vitamin D signaling in UVR induced skin cancer is that the 1,25(OH)_2_D_3_ produced in the skin under the influence of UVR provides protection against UVB and likely UVA induced tumors, a rationale supported by epidemiologic evidence that suggests benefit of low dose UVR. For example, modest increases in UVB appeared to be protective against skin cancer in a meta analysis of 10 US studies (Armstrong and Kricker, 2001), although higher doses of UVB were not protective. Moreover, no significant correlation in SCC incidence was observed in association with time spent outdoors in an Australian population study (English et al., 1998). In a multicenter European study Rosso et al. (1996) identified a threshold of 70,000 accumulated hours of sunshine below which an increase in SCC was not observed, although the threshold for BCC was lower. Thus, the clinical evidence is at least supportive if not definitive for a protective role of low dose UVB, mediated we submit via vitamin D production. Although a similar case has not been made for calcium in epidermal tumor formation, the epidemiologic data demonstrating protection against colorectal cancer by diets high in calcium and vitamin D is substantial (Garland et al., 1985; Chakrabarty et al., 2005), and as will be discussed later in this article, the role of calcium appears to be synergistic with that of vitamin D.

## Regulation of keratinocyte proliferation and differentiation by calcium and 1,25(OH)_2_D

Calcium and 1,25(OH)_2_D are critical for keratinocyte differentiation. Calcium concentrations below 0.07 mM promote proliferation, whereas increasing the extracellular calcium concentration (Cao) above 0.1 mM (calcium switch) induces differentiation. Among the changes are the translocation of proteins such as E-cadherin to the membrane to form the E-cadherin/catenin complex (adherens junctions). Proteins associated with this complex include phosphatidyl inositol 3 kinase (PI3K), various catenins including β-catenin, and phosphatidyl inositol 4-phosphate 5-kinase 1α (PIP5K1A). These proteins in this complex mediate much of the ability of calcium and vitamin D to promote differentiation (Tu et al., 2001, 2005; Xie et al., 2005, 2009; Xie and Bikle, 2007; Tu C. et al., 2008). Other important proteins whose translocation to the membrane promotes the differentiation process include the calcium sensing receptor (Casr), phospholipase C-γ1 (Plcg1), and the Src family of tyrosine kinases, the activation of which phosphorylate the catenins facilitating their binding to E-cadherin to form the E-cadherin/catenin complex. These changes then lead to the sequential induction of proteins including keratins Krt1 and Krt10 (Yuspa et al., 1989), profilaggrin (the precursor of filaggrin [Flg]), involucrin (Ivl), and loricrin (Lor). These and other proteins are cross linked into the insoluble cornified envelope by the calcium induced transglutaminase 1 (Tgm1) (Thacher and Rice, 1985; Hohl, 1990), the final step in the differentiation process.

The Casr underlies the ability of the keratinocyte to respond to calcium (Tu et al., 2004, 2011, 2012; Tu C. et al., 2008). The Casr through the scaffold protein filamin activates the RhoA pathway that in turn activates the src kinase family, which phosphorylate the catenins, facilitating their binding to E-cadherin (Tu et al., 2011). We cloned the Casr from keratinocytes (Oda et al., 1998) and subsequently developed a mouse expressing a floxed form of *Casr* (exon 7 encoding the entire transmembrane domain and intracellular portion of the gene; Chang et al., 2008; Tu C. et al., 2008). This enables us to delete the gene in the tissue of our choice, in this case the keratinocyte where we demonstrated the central role of Casr in calcium signaling within the keratinocyte and its impact on differentiation (Tu C. et al., 2008; Tu et al., 2012). Mice lacking the Casr develop a defective permeability barrier due to abnormal production of essential lipids and proteins required for barrier formation as well as a defective innate immune response. Similar abnormalities develop in mice with *VDR* or *Cyp27b1* gene deletions. Moreover, deletion of Casr also results in reduced expression of *Vdr* and *Cyp27b1* (Tu et al., 2012), likely contributing to the failure of the epidermis of Casr deficient mice to differentiate normally. On the other hand 1,25(OH)_2_D induces the *Casr* (Canaff and Hendy, 2002). Just as 1,25(OH)_2_D/VDR induces *Casr*, calcium/Casr is required for *Vdr* and *Cyp27b1* expression, demonstrating the strong interaction between calcium and vitamin D signaling in the skin with respect to differentiation. Thus, the actions of 1,25(OH)_2_D/VDR enhance the keratinocyte response to the prodifferentiating actions of calcium (Ratnam et al., 1999), whereas the effect of Casr in the expression of *Vdr* and *Cyp27b1* (Tu et al., 2012) enhances the prodifferentiating actions of 1,25(OH)_2_D (Su et al., 1994). With respect to cancer, this synergistic interaction is an important concept to which we will return.

The synergism between Casr and VDR is well-demonstrated by the joint regulation of the expression of a number of genes by calcium and 1,25(OH)_2_D including the phospholipase C (*Plc*) family members (Xie and Bikle, 1997) important for differentiation, the processing of the lipids required for permeability barrier formation (Oda et al., 2009), and the enhancement of the innate immune response via induction of toll like receptor 2 (TLR2) and its coreceptor CD14, that initiate the innate immune response in skin leading to the expression of defensins such as cathelicidin (Schauber et al., 2007). Moreover, both calcium and 1,25(OH)_2_D inhibit genes such as *Myc* (Matsumoto et al., 1990) and *Ccnd1* (Bikle, 2011) while inducing cell cycle inhibitors Cdnk1a (aka p21^cip^) and Cdnk1b (aka p27^kip^), which contribute to the antiproliferative actions of calcium and 1,25(OH)_2_D. The roles of calcium/Casr and 1,25(OH)_2_D/VDR in immune regulation as well as in proliferation and differentiation likely contribute to their roles in protection of the skin against the development of skin cancer.

## The roles of VDR and casr in cancer protection

Zinser et al. (2002) made the first clear demonstration of the predisposition to tumor formation in the skin of mice lacking VDR. They administered the carcinogen 7, 12 dimethylbenzanthracene (DMBA) to *Vdr* null mice and wildtype mice and found that nearly all the *Vdr* null mice developed papillomas, whereas few if any of the wildtype mice did. Others have confirmed these results (Indra et al., 2007). Moreover, *Vdr* null mice have also been shown to be predisposed to tumor formation following prolonged UVB exposure first by Ellison et al. (2008) and subsequently by our own group (Teichert A. E. et al., 2011). Following UVB both SCC and BCC develop, not just papillomas. Part of this predisposition to tumor formation is due to a defective DNA damage repair process (review in Bikle, 2012a). The appearance of BCC suggested that the hedgehog (HH) signaling pathway was involved, as mutations in this pathway underlie essentially all BCC (Aszterbaum et al., 1998). However, skin lacking VDR also results in BCC when β–catenin signaling is increased (Pálmer et al., 2008). Thus, we became interested in the interacting roles of HH and β-catenin signaling in tumor suppression by VDR, a subject to which I will return.

When we knocked out both *Vdr* and *Casr* in keratinocytes (^epid^*Vdr*^−∕−^/^epid^Casr^−∕−^, DKO), tumors developed spontaneously, something that we had not observed in mice in which either gene was deleted by itself (Bikle et al., 2015). In this case the tumors were SCC. Colorectal cancer provides a good model for the development of skin tumors in that abnormalities in calcium, vitamin D and β-catenin signaling have also been implicated in the development of colorectal cancer with human colorectal cancer cell lines (Chakrabarty et al., 2003, 2005; Bhagavathula et al., 2007; Liu et al., 2010; Wang et al., 2010). A frequently used model involves a mutated *Apc* resulting in increased Wnt/β-catenin signaling (Arimura et al., 2009). Activation of the Wnt/β-catenin pathway increases proliferation and reduces apoptosis, whereas inhibition of this pathway has the reverse effect (Varnat et al., 2009). As it does in keratinocytes calcium via the Casr blocks the translocation of β-catenin to the nucleus in part by increasing its binding to the E-cadherin/catenin complex in the membrane, thus blocking its transcriptional activity (Chakrabarty et al., 2003). Like the situation in keratinocytes, 1,25(OH)_2_D synergizes with calcium in these actions by inducing the expression of Casr (Chakrabarty et al., 2005), increasing the expression of the cell cycle inhibitors Cdkn1a and Cdkn1b, and inhibiting the expression of Myc, Ccnd1, survivin (Birc5), and thymidylate synthase (Tyms; Bhagavathula et al., 2007; Liu et al., 2010). In addition to its stimulation of E-cadherin/catenin complex formation, thus limiting the access of β-catenin to the nucleus, 1,25(OH)_2_D induces an inhibitor of Wnt //β-catenin signaling, dickkopf 1 (Dkk1; Pendas-Franco et al., 2008). Our studies in which *Casr* was deleted from the intestinal epithelial cell (Rey et al., 2011) demonstrated hyperproliferation in these cells. This was accompanied by increased localization of β–catenin in the nuclei signifying activation of β-catenin signaling. Again comparable to that shown in the skin, mice lacking *Vdr* in the intestinal epithelium were predisposed to carcinogen induced tumor formation (Byers et al., 2011). Whether deleting both *Vdr* and *Casr* will lead to spontaneous colorectal tumors remains to be tested.

1,25(OH)_2_D has also been shown to be protective against UVA induced oxidative induced mutations in DNA (Gordon-Thomson et al., 2012). UVA induces reactive oxygen species (ROS) such as superoxide anion (${\text{O}}_{2}^{-}$), hydrogen peroxide, and hydroxyl radicals as well as nitric oxide (NO) via stimulation of NO synthase. NO combines with ${\text{O}}_{2}^{-}$ to form peroxynitrite that causes oxidative and nitrative modifications of DNA bases such as 8 oxo 2 deoxyguanosine (D'Orazio et al., 2013). These UVA fingerprint lesions along with UVB fingerprint lesions have been found in human SCC (Agar et al., 2004). Of interest is that the protection by 1,25(OH)_2_D of the formation of these UVA fingerprint mutations may not require the VDR (Gordon-Thomson et al., 2012), and the role of calcium in their formation has not been studied.

## The hedgehog (HH) pathway in epidermal tumor formation

Activation of HH signaling has been shown to result in BCC in both animals and humans (Hahn et al., 1996), and appears also to predispose to UVB induced SCC (Ping et al., 2001). This was first demonstrated when *PTCH1* mutations were found to be the cause of the basal cell nevus syndrome (BCNS) (Gorlin Syndrome), a syndrome in which patients readily develop BCCs (Hahn et al., 1996; Aszterbaum et al., 1998). This syndrome has been reproduced in a mouse model with *Ptch1* mutations (Aszterbaum et al., 1999). Subsequently it was found that essentially all BCCs, sporadic or part of the BCNS, have mutations in *PTCH1* or other elements of the HH signaling pathway (Aszterbaum et al., 1998), and nearly all BCC in humans or mice overexpress Ptch1 or one of the other components of the HH pathway (Tojo et al., 1999; Bonifas et al., 2001). A drug, vismodegib, targeting the HH pathway, has recently been developed as the first effective non-surgical treatment for advanced BCC (Sekulic et al., 2012; Tang et al., 2012).

In the basal state Ptch1 inhibits the function of smoothened (Smo), like Ptch1 a membrane protein. Smo, in the inhibited state, keeps the Gli family of transcription factors bound to suppressor of fused (Sufu) within the cytoplasm, thus limiting their translocation to the nucleus (Barnfield et al., 2005; Svärd et al., 2006). Sonic hedgehog (Shh) binding to Ptch1 reverses its inhibition of Smo enabling the release of the Gli factors from Sufu and their entry into the nucleus. Gli1 and 2 are the main actors in gene transcription (Mimeault and Batra, 2010). Their transcriptional activity includes the increased expression of other components of the HH pathway, as well as anti apoptotic and cell cycle factors including Bcl2, Ccnd1,2, E2f1, Cdc45, thus promoting proliferation, while suppressing genes associated with keratinocyte differentiation including the VDR (Grachtchouk et al., 2000; Nilsson et al., 2000; Regl et al., 2002, 2004a,b). When Gli1, Gli2, or Shh are overexpressed in basal keratinocytes, BCC develop (Oro et al., 1997; Grachtchouk et al., 2000; Nilsson et al., 2000). Similarly human keratinocytes overexpressing Shh develop BCC like lesions when grafted unto nude mice (Fan et al., 1997).

*Vdr* null animals overexpress elements of the HH signaling pathway in their epidermis (Teichert A. et al., 2011), although they show reduced expression in the cycling portion of their hair follicles (Teichert et al., 2010). Consensus sequences for vitamin D response elements (VDRE) in the promoters of Shh, Ptch1, Ptch2, Gli1, and Gli2 have been identified (Reddy et al., 2004; Wang et al., 2005; Pálmer et al., 2008; Luderer et al., 2011), and we (Teichert A. et al., 2011) have demonstrated that 1,25(OH)_2_D inhibits their expression in a VDR dependent fashion. Moreover, vitamin D *per-se* has been shown to suppress Smo (Bijlsma et al., 2006; Tang et al., 2011), presumably by a non-genomic mechanism. These findings in the epidermis stand in stark contrast to the role of VDR during HF cycling where the unliganded VDR appears to promote the ability of HH signaling to initiate early anagen (Lisse et al., 2014).

## β-catenin signaling in epidermal tumor formation

Benign hair follicle tumors, pilomatricomas or trichofolliculomas, result from over activation of the wnt/β-catenin pathway (Gat et al., 1998; Chan et al., 1999; Xia et al., 2006). However, this depended on the VDR status of the animal. Pálmer et al. (2008) demonstrated an interaction between VDR and β-catenin in transcriptional regulation. They found putative response elements for VDR and β-catenin /LEF1 in a number of genes including those of the HH signaling pathway. Moreover, an analog of 1,25(OH)_2_D could block the formation of these hair follicle tumors induced by overactivation of the wnt/β-catenin pathway. On the other hand, mice lacking VDR developed BCC rather than the more benign trichofolliculomas when the wnt/β-catenin pathway was overactivated. In humans trichofolliculomas were found to have high nuclear levels of both β-catenin and VDR, whereas BCC have high levels of β-catenin but low levels of VDR (Pálmer et al., 2008). These data indicate the importance of the interactions between VDR and β-catenin in the hair follicle and in the epidermis in both mice and humans. As in HH signaling, these interactions are complex in that VDR is required for β-catenin activation in the hair follicle (Lisse et al., 2014), such that when β-catenin transcriptional activity is prevented as in the VDRKO, hair follicle formation is blocked (Huelsken et al., 2001). On the other hand suppression of β-catenin transcriptional activity by VDR in the epidermis appears to be protective with respect to tumor formation (Wei et al., 2007).

As noted earlier both the Casr and VDR are required for the formation of the E-cadherin/catenin complex, which is not only required for the differentiation of the keratinocyte, but by keeping β-catenin bound to the membrane prevents its nuclear translocation and activation of genes promoting proliferation. Loss of the E-cadherin/catenin complex is a well-known marker of malignant transformation in a number of epithelial cells (Ghahhari and Babashah, 2015). Moreover, we (Tu et al., 2007; Tu C.L. et al., 2008) have shown that deletion of the Casr from keratinocytes reduces their stores of calcium and blocks their response to extracellular calcium (Cao) including the formation of the E-cadherin/catenin complex.

**Figure 1:**
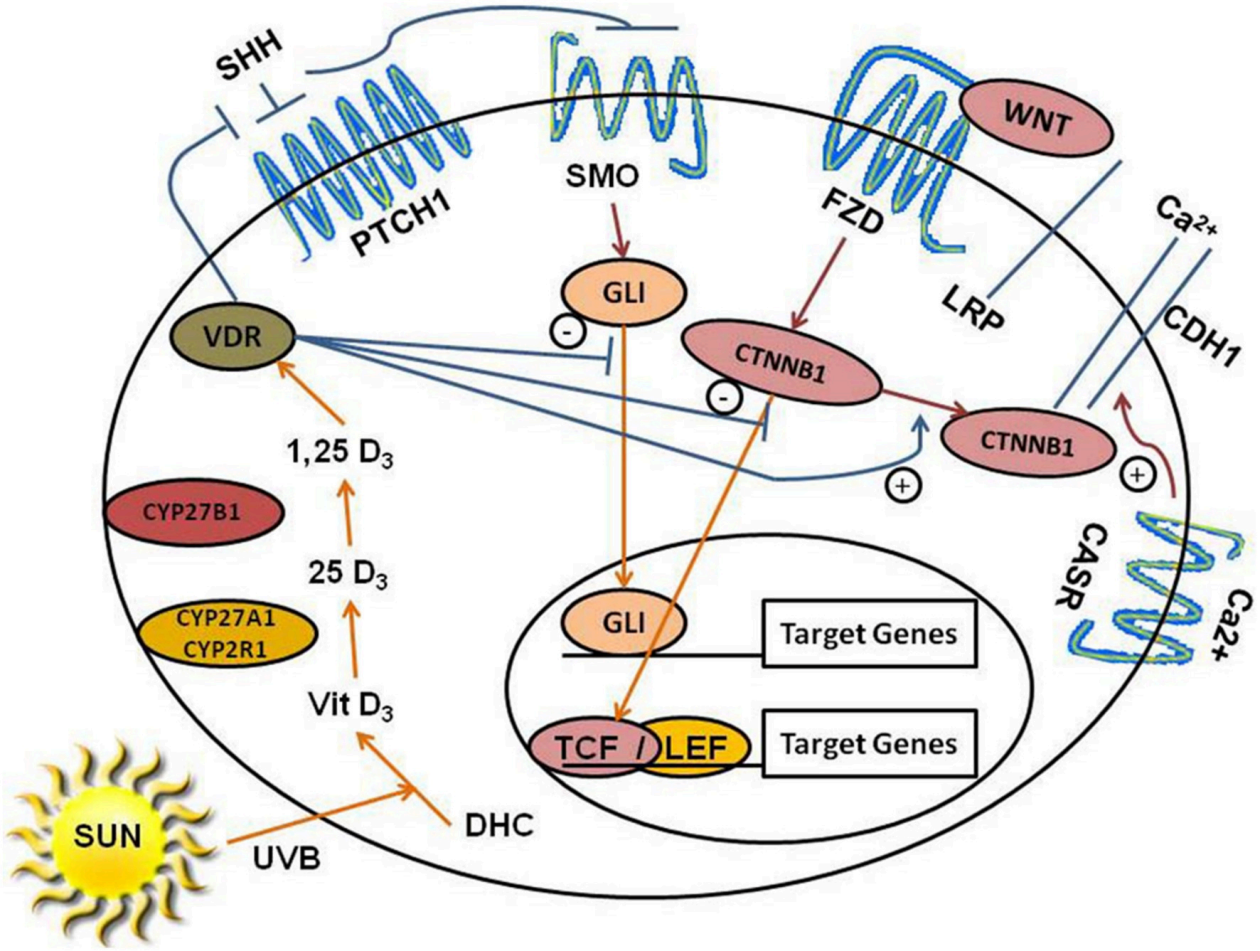
**Regulation of HH and Wnt/β-catenin signaling by 1,25(OH)_**2**_D/VDR and calcium/Casr**. The keratinocyte expresses VDR and is capable of making its own 1,25(OH)_2_D_3_ from the vitamin D_3_ produced from 7-dehydrocholesterol (DHC) under the influence of UVB, as it has both Cyp27a1/Cyp2r1 (which convert vitamin D_3_ to 25OHD_3_) and Cyp27b1 [which converts 25OHD_3_ to 1,25(OH)_2_D_3_]. The keratinocyte also expresses the calcium sensing receptor Casr required for calcium induced differentiation. 1,25(OH)_2_D/VDR suppresses *Shh* and *Gli1* expression, inhibiting the HH pathway in keratinocytes. 1,25(OH)_2_D/VDR binds CTNNB1(β-catenin) and increases CDH1(E-cadherin) levels in the plasma membrane reducing the amount of β-catenin available for binding to TCF/LEF in the nucleus limiting its transcriptional activity. Calcium acting through its receptor is required for the formation of the E-cadherin/catenin complex in the plasma membrane. In combination these actions reduce the proliferative actions of Shh and Wnt/β-catenin signaling in keratinocytes, limiting their ability to induce tumors in the skin.

The β-catenin and HH pathways interact (Bienz, 2005; Pálmer et al., 2008). Using a constitutively active *Smo* [*Gt(ROSA)26Sor*^*tm*1(*Smo*∕*EYFP*)*Amc*^] in keratinocytes to induce BCC, two groups (Yang et al., 2008; Youssef et al., 2012) found a rapid increase in genes of the Wnt/ β-catenin pathway. *Dkk1* overexpression or deletion of *Ctnnb1* prevented the development of BCC. Both HH and Wnt/ β-catenin pathway constituents were found to be over expressed in a series of human BCC (Youssef et al., 2012). Putative β-catenin /LEF1 response elements as well as VDRE mentioned earlier have been found in a number of HH pathway genes (Pálmer et al., 2008), which unlike the VDRE appear to be stimulated by activated β-catenin with an increase in *Shh* expression (Schneider et al., 2010). The role of VDR in the regulation of these two pathways is shown in Figure 1.

## Long non-coding RNAs (lncRNA)

Approximately 20,000 protein-coding genes are encoded by the human genome. This represents less than 2% of the total genomic sequence. That said 90% of the genome is actively transcribed without protein coding potential (Mercer et al., 2009). These non-coding transcripts are arbitrarily divided into short and long non-coding RNAs with small non-coding RNAs defined as less than 200 bases, including tRNAs, microRNAs, and small nuclear (snoRNAs), and long non-coding RNAs (lncRNAs) as those with lengths larger than 200 bases, some over 100 kb in length (Gibb et al., 2011). Much of the transcriptome is comprised of lncRNAs (~80%; Mercer et al., 2009), and over 23,000 have been identified so far. Like mRNAs they are spliced and contain polyadenylation signals, (Mattick, 2011). Many regulatory processes are controlled by lncRNAs including embryonic pluripotency, differentiation, and body axis patterning, promoting developmental transitions (Mattick, 2011; Batista and Chang, 2013). LncRNAs can influence the epigenetic programs of the transcriptome through their regulation of histone modifications (Spitale et al., 2013). Of particular relevance to this review is that lncRNAs regulate cancer development through a number of effects on tumor cell proliferation, blocking growth suppressors, inducing angiogenesis, and promoting invasion and metastasis (Gibb et al., 2011; Gutschner and Diederichs, 2012; Li et al., 2013).

We (Jiang and Bikle, 2014) evaluated the potential role of lncRNAs in VDR protection against skin tumor formation using *in vitro* cultured mouse keratinocytes and an *in vivo* mouse model, comparing cell or mouse epidermis from wildtype or *Vdr* null animals, in an array containing 90 well-annotated mouse lncRNAs. We found increased expression of several well-known oncogenes, including *H19, HOTTIP*, and *Nespas*, and reduced expression of tumor suppressor genes such as *Kcnq1ot1, lincRNA-p21* in VDR deleted keratinocytes whether from cultured cells or epidermis. These results point to an additional mechanism for protection by VDR against skin cancer formation that we are in the early stages of exploring. Whether concomitant *Casr* deletion will amplify these results has not yet been determined.

## Conclusions

Both calcium and vitamin D signaling through their respective receptors Casr and VDR are important for the normal functions of the skin. In this article we have focused on their roles in tumor development when such signaling is disrupted by deletion of the receptors. At this point we have not observed tumor formation in the skin of mice lacking only the Casr, but have observed that mice lacking both Casr and VDR develop tumors of greater malignancy and spontaneity than seen in mice lacking only the VDR. Analyses of gene expression in the epidermis from mice lacking one or both of these receptors clearly demonstrate the synergism between VDR and Casr in gene regulation including genes involved with cancer such as the E-cadherin/catenin pathway that plays such an important role in the epithelial/mesenchymal transition leading to tumor formation. We have focused on two pathways, HH and Wnt/ β-catenin, that are regulated by VDR and Casr, and that are plausible participants in epidermal tumor formation when their regulation is disrupted. In addition we have introduced a new mechanism, alterations in lncRNA expression toward an oncogenic profile, when VDR is deleted. No doubt other pathways will emerge that contribute to a greater or lesser degree to the predisposition of the epidermis lacking VDR and/or Casr to tumor formation.

## Author contributions

DB is the senior author of the paper. He wrote the text and ran the laboratory where the work was done. YJ performed the experiments showing the development of SCC in mice lacking both the VDR and CaSR, as well as the LncRNA profiling. TN assisted with many of the technical aspects of the experiments described in recent publications. YO provided much of the data for the publications involving VDRKO mice. CT developed the CaSRKO mouse and did most of the studies with this mouse.

### Conflict of interest statement

The authors declare that the research was conducted in the absence of any commercial or financial relationships that could be construed as a potential conflict of interest.
